# Determinants of Child Health Behaviors in a Disadvantaged Area from a Community Perspective: A Participatory Needs Assessment

**DOI:** 10.3390/ijerph15040644

**Published:** 2018-03-31

**Authors:** Manou Anselma, Mai Jeanette Maidy Chinapaw, Teatske Maria Altenburg

**Affiliations:** Department of Public and Occupational Health, Amsterdam Public Health Research Institute, VU University Medical Center, van der Boechorststraat 7, 1081 BT Amsterdam, The Netherlands; m.chinapaw@vumc.nl (M.J.M.C.); t.altenburg@vumc.nl (T.M.A.)

**Keywords:** participatory, health, children, socioeconomic

## Abstract

Children from disadvantaged areas are hard to reach for interventions aimed at promoting healthy lifestyles. We conducted a participatory needs assessment, in which researchers collaborated with a community in a disadvantaged area in Amsterdam to gain an understanding of the health-related issues of children within this community. Qualitative data was collected through: three to four participatory group meetings with three groups of 9–12-year-old children (*n* = 5–9 per group); nine interviews with professionals working with youth; two interviews with parents and their children; and informal meetings including 31 parents. All transcriptions or summaries were coded and analyzed. Childhood overweight/obesity was indicated as the main health issue. A lack of physical activity and unhealthy dietary behavior were identified as the main risk factors, with underlying determinants such as culture, habits, finances, and social norms. Identified needs included more supervised, low-priced sports activities at a nearby location and more education on adopting a healthy diet. Our participatory health needs assessment resulted in a comprehensive overview of the most relevant risk factors and determinants of childhood overweight/obesity and needs from the community’s perspective. This knowledge aids in the development of better tailored, and thereby potentially more effective, interventions.

## 1. Introduction

Children living in families with a lower socioeconomic position (SEP) generally have more health problems than children from families with a higher SEP. Children from lower SEP families have for example more mental health problems, higher mortality rates, higher prevalence of obesity, and physical and social problems [1,2,3,4,5,6,7]. Therefore, improving healthy behavior is high on the agenda in disadvantaged areas in The Netherlands.

Due to modernization and urbanization, many societies are evolving into obesogenic living environments for children [8]. In today’s society, children play videogames inside the home rather than actively play outside, and there is a growing availability of mechanized transportation and inexpensive high-caloric foods [8,9,10,11]. These are just a few of the examples that put today’s children at a higher risk for engaging in unhealthy behaviors.

Health issues such as overweight and obesity are largely caused by unhealthy behavior, and both overweight and unhealthy behaviors are disproportionately divided among socioeconomic groups [3,4,5,6,7]. In Western countries, more children from low SEP families are overweight or obese [12,13]. Therefore, interventions trying to promote healthy lifestyle behaviors tried to specifically target this vulnerable group [14,15,16,17]. However, effectively reaching this vulnerable target group in interventions is very challenging [18,19,20,21]. A clear understanding of the needs, preferences, and characteristics of the target group is essential for the development of an effective intervention [18,22,23]. However, mapping all this information is time-consuming and is therefore not always included in needs assessments and rarely conducted in collaboration with the target group [24]. Yet a thorough needs assessment should include the community’s perspective [22,25]: how would they describe the problems occurring in their neighborhood, how, in their opinion, does this situation influence a healthy lifestyle for children and how would they themselves deal with this problem?

A participatory needs assessment differs from a regular needs assessment in the sense that it is bottom-up. Not only is research done ‘on’ a certain community, but at the same time ‘with’ the community [26]. Similar to a regular needs assessment, the focus is on gaining knowledge about the community’s needs. For a participatory needs assessment, researchers collaborate with the community to gain the above-mentioned knowledge [22]. As a result, the community benefits directly from this participatory process as they are involved, educated and mobilized [26]. Community members as well as professionals are included in the research process in a partnership with academic researchers to understand the health needs through the eyes of the community. In this process, ideas can also be proposed concerning how these health needs could be answered [27,28]. Therefore, such a participatory needs assessment provides essential input for the development of a childhood obesity prevention intervention targeting this community [24].

The current study therefore aims to conduct a thorough participatory needs assessment to gain knowledge about the health issues and needs of children in a low-income community in Amsterdam. This needs assessment will be used as input to develop interventions aimed at promoting health behaviors of children in the community. By conducting this participatory needs assessment, the interventions that will be developed in the future can be tailored to the specific needs and interests identified by the community itself.

## 2. Materials and Methods

### 2.1. Recruitment

This qualitative study took place in a disadvantaged neighborhood in Amsterdam. Because of the high percentage of families with children (37%) and the relatively high number of people living in poverty, the neighborhood was defined as a high-priority area [29]. In the neighborhood where this study took place, 50% of the people were from a non-Western background [30]. The largest ethnic groups were from a Dutch, Moroccan, Surinamese, and Turkish backgrounds (40.5, 11, 11, 7%, respectively). Of the people in the neighborhood, 24% were under the age of 18, and 27.5% of these youngsters were growing up in a poor household (an income up to 110% of the Dutch minimum standard and capital below the social welfare limit). Certain areas of the neighborhood have been described by the city council as having multiple socioeconomic issues such as poverty, unemployment and debts. Health and self-perceived health were rated as poor, with, for example, 61% of adults being overweight or obese, 27% of the 10-year-olds, and 16.8% of the 5-year-olds [30].

We chose to work together with primary schools, to reach as many children from the community as possible. All four primary schools in the selected area were contacted by the municipality and informed about the project, including the needs assessment and subsequent development and implementation of interventions. After this initial contact, the researcher contacted the schools by phone. Three schools volunteered to participate, for the fourth school it was too short notice.

At the three participating schools, all children between the ages of 9 and 12 (i.e., children in the three highest grades of primary school) were invited to join a participatory research group (PAR group, a group of children partaking in participatory meetings). We focused on children aged 9–12 years as they have been found to be able to participate in such a research group [31]. As engaging in healthy behaviors received a lot of attention at the participating schools, children already had a lot of knowledge and ideas on this topic. All children received an information letter for themselves and one for their parents, the latter including an informed consent letter. At two schools the researcher got the opportunity to give information about the project in the classrooms. If children wanted to participate, one of their parents had to sign the informed consent form and return it to the researcher. In the PAR groups, there was room for six to eight children; if more than eight children signed up, eight children were randomly selected.

In addition to the children themselves, we aimed to include parents of 9–12-year-old children in this neighbourhood, as parents play an important role in children’s lives. Moreover, we aimed to include professionals working with 9–12-year-old children in the community (e.g., school staff, youth workers, social workers, policy makers). Parents were recruited through a sports camp for overweight children as well as through schools. Through the sports camp, parents (*n* = 5) living in the study area were contacted by phone and asked to participate in an interview together with their child. One father and one mother agreed to participate. Additionally, all parents of children aged between 9 and 12 attending the three primary schools in the selected area received a letter through the schools, inviting them to participate in participatory group meetings for parents. Unfortunately, no parents signed up. Therefore, we gathered information through informal meetings with mothers visiting the community center. All mothers were welcome to join the discussion. We emphasized that we were focusing on children between the ages of 9 and 12, so parents with much younger or older children participated less in the discussions or spoke about general experiences in the community.

In this community center, women, mostly mothers, came together for different events such as a free hairdresser or sports sessions. The researcher informed the women who visited the community center about the study. Although the women agreed to participate in informal (group) interviews, they did not want to be recorded or spend extra time on participation because they said they were busy.

The municipality provided contact information of relevant and experienced professionals working with 9–12-year-old children in the neighborhood (such as health professionals, youth workers, and teachers), leading to purposeful sampling. Professionals were contacted by e-mail (*n* = 30) and were invited to participate in a group interview or an individual interview, as they preferred. All respondents (*n* = 9) preferred an individual interview, as this was easier for them to schedule.

### 2.2. Procedures

Health needs of children in the community were obtained by working together with the community. All meetings and interviews were led by the primary author M.A.; she is the project supervisor with experience in participatory research. All participants were aware of the goal of the study and the role the researcher had, whom they did not know before. The researchers started all meetings by asking which health problems children in the community experience. From the demographic information from the municipality and local health services (GGD), the initial interviews with professionals and the first parent meeting, it became clear that the focus was on physical activity and dietary behavior. Therefore, additional child and parent interviews were scheduled with participants of a summer camp aimed at a healthy lifestyle.

#### 2.2.1. Participatory Meetings with Children

Participatory meetings (60 min) were held with the PAR groups at the three schools. At school one, the group consisted of six participants (four girls, two boys) who met four times after school hours; at school two, the group consisted of five participants (one girl, four boys) who met three times after school hours; at school three, the group consisted of nine participants (seven girls, two boys) who met three times during school hours. All parents gave informed consent to their child’s participation and to audio recordings. After each meeting, the recordings were transcribed and used as input for the next meeting.

In the first meeting, an introduction of the participants and facilitators took place as well as an introduction to the project. At each meeting, two facilitators were present. The facilitator who led the meeting was an academic researcher (M.A.); the other facilitator was a male social worker. The social worker started each meeting with an icebreaker, and assisted the other facilitator and the children throughout the meetings. In the first meeting, the children were asked what they thought health was, and what the topic encompassed according to them. This was done to get them thinking about the topic, and to ascertain whether they were able to discuss the topic in the participatory meetings. The children were then asked what kind of health issues they experienced in their community and what they thought the causes were. In the second meeting, the children could pick a topic/cause from the first session that they wanted to explore. Children, for example, picked low participation in sports or low consumption of fruit. The facilitator guided them in how to formulate a research question, how to select a suitable research method, how to collect data and how to process the data. In the last session, children’s findings were discussed. Together with the facilitators, the children analyzed their own data and formulated conclusions. Each group made a poster to present the results of their research and the ideas they had to improve the current situation. Children, for example, interviewed peers on sports participation, found out this was low and came up with the idea to organize extra sports sessions at school.

#### 2.2.2. Meetings with Parents

The researcher informally interviewed mothers in the community center about their life in the community and the issues they encountered, in general, and focused on the health of their children. Additionally, they were asked about their perspectives on underlying causes of their children’s health problems, initiatives that target these issues, and ideas for new initiatives. One short brainstorm session (15 min) took place with six mothers after they participated in a sports class. In this brainstorm session, the mothers discussed what they missed in the community for their children. Additionally, two group discussions (45 min) took place, each with 10 mothers who visited the hairdresser in the community center. Four mothers participated in both group discussions. Additionally, there were five individual discussions (15 min) with mothers who visited the hairdresser. During these individual discussions, the mothers often asked the opinion of others who were present, which resulted in input from five other mothers. Because of language difficulties, the researcher could not communicate directly with all mothers, but the mothers translated for each other and discussed what they wanted to express. As the discussions took place in an informal setting, the topic of health behaviors could be discussed in an informal and open atmosphere. This was important for the mothers, as talking about their home environment appeared a sensitive subject for many of them. Hence, none of the conversations were recorded.

#### 2.2.3. Interviews with Professionals

The interviews with professionals were semi-structured and lasted 30–60 min. Nine interviews were conducted with professionals working in the community. All participants agreed to the audio-recording of the interview. After the interview, the recording was transcribed and a summary of the transcript was returned to the participant for verification. Three professionals worked for a school program stimulating healthy school environments; two worked for the municipality in sports and/or child development; one was a physical education teacher; one was the manager of a community center; one was a youth mentor; and one was a social worker. See Box 1 for the interview guide.

Box 1 **Questions for interview with professionals.**

1. What is your professional background and what kind of work do you specifically do?
2. What kind of health-related problems do you see with children in this community?
3. Can you think of underlying causes for these problems? Can you categorize and rank them according to your opinion?
4. How do families handle these problems?
5. What kind of solutions can you think of to solve these problems?
6. Are there already initiatives in the community that are trying to deal with these problems?
7. Are these initiatives working and why (or why not)?
8. You just ranked the problems. If you think for the families, which problems do you think they would want to tackle first?
9. What can be done so that existing or new programs suit the needs of the families better?
10. Do you have any suggestions for our study?

#### 2.2.4. Interviews with Children and Parents

Two semi-structured interviews of about 30 min were held with children who participated in a summer camp for children with overweight, and their parents. The interviews were designed to obtain more information about their motivations to participate in an intervention targeting healthy behavior and how they thought other families could be motivated to improve their lifestyle. We first explained that we wanted to learn from their experience through the interview. The first interview was with a girl of 11 years old and her mother. The second interview was with two brothers of 9 and 11 years old and their father. Children and parents verbally consented to the interview. No audio-recording took place as both parents did not feel comfortable being recorded. See Box 2 for the interview guide.

Box 2 **Questions for interview with children and parents.**

1. Why did you decide to participate in the summer camp?
2. What did your dietary behavior look like before you joined the camp?
3. Did you already participate in sports before you joined the camp?
4. What did you learn at the camp?
5. What would you like to improve in the neighborhood, and how, so you can have a more healthy lifestyle?
6. What do you think holds other families back from having a healthy lifestyle?
7. Can you name three reasons why children do not exercise enough?
8. Can you name three reasons why children do not have a healthy diet?

### 2.3. Data Analysis

The interviews with professionals and the participatory meetings with children were audio-recorded and transcribed. Summaries of the interviews were sent to the professionals for verification, and the key points from the participatory meetings were verified at the beginning of the next meeting. During the meetings with mothers and during the interviews of camp participants, notes were taken and summarized afterwards [32,33]. Additionally, experiences of the facilitators during the participatory meetings with children were noted. The researcher and other facilitators sat together after every meeting to discuss their notes. The transcriptions and summaries were coded in MaxQDA using open-coding [34]. Codes, and relations between codes and categories, that were identified from these texts were verified in later meetings with the participants [35]. Table 1 depicts the main coding scheme. The three main themes have been broken down into categories and again into subcategories [34]. The interaction within and between these categories and subcategories was iteratively analyzed, whereby additions and changes could be made in order to create a complete and practically applicable overview.

## 3. Results

In all interviews and meetings, the same question was asked: “What are the main health problems that children in this neighborhood encounter?” Professionals answered this question with unhealthy behavior and overweight, children and parents answered this question predominantly with unhealthy behavior. The subsequent interviews and meetings therefore focused on the identified behaviors, to further specify underlying determinants and suggestions to improve children’s health.

### 3.1. Causes of Unhealthy Behavior

The two main unhealthy behaviors that were mentioned by children, parents, and professionals were insufficient physical activity and unhealthy dietary behaviors—specifically consumption of sugared drinks and unhealthy snacks—for which many underlying causes were mentioned.

#### 3.1.1. Insufficient Physical Activity

An important topic mentioned by all stakeholder groups was **safety**: people living in the community do not feel safe in the neighborhood. This is the main reason that parents do not let their children play outside (mothers). One factor contributing to the perception of parents that their neighborhood is unsafe were groups of youngsters hanging around in the neighborhood and causing trouble. There was a registered youth gang in the area who “[…] terrorize the neighborhood: they throw stones, sticks...” and set things on fire (professional). Another factor contributing to the perception that the neighborhood is unsafe was the soft drug shop situated in the center of the neighborhood. The soft drug shop is located on a square where also two of the community centers are located plus one of the primary schools. The children said that the people who leave the soft drug shop are strange and disgusting. One of the mothers said that they throw a lot of trash in her garden and that they roam around the streets. Another mother said that people from the soft drug shop hang around in the alley near her house and that she does not feel safe going through that alley to reach her house. To deal with this issue of safety, activities should be organized at a safe, well-known location and should be supervised (camp parents, mothers, professionals). Safety also played a part in the topic of **distance**. As the community is perceived as being unsafe, parents do not like their children walking around by themselves (mothers, professionals). The district of which the community is a part of, offers quite a lot of sports activities. However, activities organized in the neighborhood but at a different school, may already be perceived as being too far by children and/or parents. “What is too far? If they have to go by bicycle, they don’t come” (professional). This was not only because of safety reasons, it also related to the comments of children and parents who said that they wanted more activities close to their home or school, because it was a barrier for parents to bring their children to an activity. Children were not allowed to go by themselves so if their parents could not bring them, they could not go.

**Finances** were an underlying cause for both inactivity and unhealthy diet (next section). Both were in general not a priority for families with a low income (mothers, camp parents, professionals). Moreover, some parents could not afford the costs for a sports club membership. In The Netherlands, the Youth Sports Fund (www.jeugdsportfonds.nl) is a fund where families living below a certain income can apply for payment of a sports subscription. Unfortunately, many parents do not know about this fund (**father camp**); think they do not qualify for the fund (**mothers**); have an income just above the minimum qualification; feel ashamed to apply for the fund; or the amount that they can request is not enough for the sports subscription (and transport and required gear) (mothers, camp parents, professionals). Related to this topic is that professionals stated that parents have **insufficient knowledge**: parents are unaware of the negative effects of physical inactivity on children’s physical and motor development, and parents are unaware of the (financial) possibilities of joining sports facilities/clubs.

**Culture** was also mentioned in relation to both inactivity and unhealthy diet. For many ethnic groups in the community, playing outside is not a habit (professionals). In most seasons, parents think it is too cold and that their children will get sick if they play outside (children, mothers, camp parents). In the interviews with the children who had participated in the summer camp, the children and one father mentioned that inactivity is also related to family **habits**. If, for example, parents are not in the habit of participating in sports activities, they also do not stimulate their children. Also, when parents are busy and perceive a lot of stress, they do not feel like making time to bring their children to practice (father camp). “But once they start, they realize how much fun sports is for their children and they will make it work” (father camp). Children and professionals indicated that when children have **overweight/obesity**, they do not like to participate in sports because they are bullied by others or cannot participate because of their physique. This shows the importance of prevention of unhealthy habits and overweight.

#### 3.1.2. Unhealthy Dietary Behaviors

Children, parents and professionals mentioned many underlying causes for an unhealthy diet. Four of them coincide with causes of inactivity, i.e., culture, habits, finances and insufficient knowledge. Firstly, **culture**: as Moroccan women from the community explained, when you visit someone, you always expect that there is a large amount of food. Most of the traditional dishes are not very healthy and contain a lot of sugar or fat. “When you don’t serve a lot of (Moroccan) food for your guests, it is considered inhospitable and ‘cheap’” (mothers, children). Mothers mentioned their interest in increasing their knowledge about healthy cooking and healthy dishes from other cultures.

Secondly, resistance to change **habits** in general was mentioned as a problem. Children and parents who participated in the summer camp said that many children are used to eating unhealthily and therefore do not want or like anything else. Setting an example as a parent is very important for children to create healthy habits (children, professionals).

Thirdly, **finances** have been mentioned as an underlying cause for unhealthy diet. Especially professionals working with families mentioned that for families who have little money to spend, fruit and vegetables are considered expensive, so they do not eat it daily. In addition, a healthy diet is not one of their priorities as they for example have to pay rent, debts and have to provide for an extended family (mothers, professionals).

This relates to the fourth coinciding topic, i.e., **insufficient knowledge**. Parents are not aware of how important a healthy diet is (professionals), what a healthy diet consists of (children, professionals), and the available inexpensive and healthy options (mothers, camp parents, professionals). Furthermore, not all parents have adequate nutritional knowledge. Most parents know that vegetables and fruits are healthy, “but not all parents know how unhealthy puffed pastries or energy drinks are” (professional). One of the children also gave a good example: “five cans of Red Bull is not normal, one a day is enough”. This shows that some children drink a large amount of energy drinks, but also that even when children understand that it is not healthy, they think that drinking energy drinks every day is alright. Additionally, professionals indicated that it seems difficult for children and parents to think about long-term effects. If for example the easy solution is to get fast food instead of preparing a meal, parents do not consider the possible effects on their child’s weight.

Another underlying cause mentioned for an unhealthy diet is that children have a lot of **decisive power**. If they for example do not like what is served for dinner, many parents make something else (mothers, camp parents, children). This is how children avoid eating vegetables. Also, children can serve themselves a second plate of food (mother camp) or get crisps whenever they want (mothers, camp parents). Parents do not necessarily like or encourage it, but allow it. What often happens, is that parents give small change to the children (one or two euros) and the children can buy whatever they want (children, mothers, camp parents, professionals); often snacks and energy drinks. Youth workers noted that many youngsters do not eat dinner at home because parents do not cook, or they just want to hang out with their friends (professional). This results in eating snacks or fast food for dinner. “If I give you two euros and tell you to go to the supermarket and buy as much as you can, then yeah what are you going to get? Then you buy a can of energy drink for 13 cents and big bags of crisps for 99 cents” (professional). When children can decide for themselves what they buy, they buy food and drinks that are perceived as cool: sugared beverages and snacks. Children mentioned that you do not want to be seen eating fruit or vegetables, because it is not cool. Therefore, they preferred to bring sugared beverages and snacks to school and bought these when they were with their friends.

Lastly, a topic mentioned by all groups was **marketing of unhealthy food**. There is a lot of unhealthy food available everywhere—at supermarkets, snack bars, and small shops—and it is often cheaper than healthy food. Food packaging influenced choices too, many of it misleading people into thinking the product is healthy. For example, packaging state 0% fat, but the product actually contains a lot of sugar.

### 3.2. Solutions

Based on the factors that children, parents and professionals consider as causing unhealthy behaviors, all participants came up with ideas to improve children’s health. Children, parents, and professionals said that there should be: (1) more organized physical activities; and (2) education about buying healthy products and preparing healthy meals.

#### 3.2.1. More Organized Physical Activities

According to children, parents, and professionals, there are a number of prerequisites for children to engage in regular physical activity (i.e., sports and play). The most important one mentioned by all participants was that there should be a **continuous activity program** for children that is offered throughout the year instead of the many short programs that are currently offered. Additionally, a limited number of children can enroll in the afterschool programs. Children, parents and professionals agreed that there should be a program that allows all children to be physically active throughout the year.

“I used to play soccer via school, but that stopped. Now I’m not enrolled anywhere.” (child, PAR group). 

“I enrolled for basketball, but I wasn’t selected.” (child, camp interview). 

“I think we have to stop with short-term projects. That you don’t have a new program every year. Not one month, or on an ad hoc basis. You know, that just doesn’t work.” (professional).

In addition, children, parents and professionals indicated the importance of organized physical activities being **affordable**. As mentioned before, many families do not have a lot of money to spend on extra activities (mothers, camp parents, professionals).

Because of the problems with transport and safety, **more supervised activities** should be organized **nearby** school or home. When an activity is supervised, parents let their children participate more easily. However, it should be organized nearby, otherwise children are not allowed to go or do not have transport. The children came up with the idea to organize a sports day at school once a week or once a month. They also mentioned they should motivate each other to play outside and change the physical environment to stimulate active behavior, for example by building new playgrounds. Most mothers also liked to exercise more, but in a safe environment with no men present and at times that are convenient for them.

#### 3.2.2. Education about a Healthy Diet

To increase knowledge about a healthy diet, children proposed education about a healthy diet at school, but would also like to learn more in practice, for example through cooking classes. Parents would like education for their children, but said that they themselves already know what is healthy and what is not. However, professionals said that especially parents need education because there are a lot of misconceptions, for example, about what exactly is healthy and unhealthy, and that healthy food is always more expensive. This is a topic where much can be gained. To get parents to attend such an educative meeting, it is important that it is organized at a safe location close to home or school and combined with a fun activity which makes it less formal (professionals). Mothers showed their interest in cooking classes, which could be a suitable activity to learn about preparing healthy meals in a practical and fun manner (children, mothers, professionals). When organizing these cooking classes, it should be considered that a lot of people in the community are not fluent in Dutch and that some are illiterate ([29], professionals, mothers). One of the professionals from the community for example said that at meetings at school a lot of parents do not understand everything that is being said. Mothers acknowledged having problems with the Dutch language.

## 4. Discussion

Our participatory needs assessments aimed to gain knowledge on the health issues and needs of children in a low-income community in Amsterdam. Children, parents, and professionals identified child overweight and unhealthy behaviors as the main health problems, with unhealthy dietary behavior and insufficient physical activity as underlying risk factors. For these underlying risk factors, context-specific determinants were identified, and participants came up with possible solutions. All stakeholder groups indicated that more education is necessary on what a healthy diet consists of, but only in a fun and practical way, for example through cooking classes. All groups also indicated that to promote physical activity, physical activities should be continually organized at a nearby location, for a low price, and that the activities should be supervised. Involving children in the development of intervention strategies taking into account these characteristics is the goal of our project at large.

To reflect on the results, it is interesting to see if the results can be placed in an ecological model. The ecological model of Davison & Birch (2001) reviews three risk factors for obesity: physical inactivity, unhealthy nutrition, and sedentary behavior, and at three levels. The first level deals with child characteristics; the second describes parenting styles, family characteristics and peers; the third level entails the community, demographics, and societal characteristics. All three levels are touched upon in our needs assessment. At the first level, child preferences and knowledge were discussed. For example, children mentioned food that they like, and sports they enjoy, and shared knowledge they have about healthy nutrition. There were also children who said that some children do not like sports and that some children are too overweight and therefore do not participate in sports. Parents mentioned, among other things, that their children did not like vegetables and preferred videogames over playing outside. At the second level, the decisive power of children over their parents, family habits, peer pressure, and rules have been mentioned as creating multiple barriers. Parents for example prepare a different meal than planned if children do not like the food or have limited time to accompany their children to a sports activity. At the third level of the ecological model, culture, norms, finances, the built environment, and transport were mentioned as influencing children’s behavior. The added value of our study lies in the deeper understanding of the local context.

Thus far, needs assessments for obesity prevention studies are typically based on a literature study or existing quantitative data with little qualitative data from the specific target group [15,36,37]. Such needs assessments also conclude that physical activity and dietary behavior should be targeted, however little information on the context of these behaviors is provided. Going in-depth into the context-specific determinants of these behaviors is often not included in the needs assessment. The reasons for this could be a broader target population—i.e., 100 schools or multiple neighborhoods—for which it is difficult to develop one intervention that fits the entire target population, or time limitations. This paper focused on children from one neighborhood, where these context-specific determinants were identified. This paper is part of a larger project, in which this information enables better tailoring of intervention strategies matching the particular needs of children in this neighborhood, or even suitable per school. Our needs assessment also stands out because it includes perspectives of not only professionals, but also parents and, most importantly, the children themselves. Because children themselves identified potential determinants, our results provide the most relevant determinants for children’s health behaviors from the child perspective. This supports the development of solutions that are applicable to their specific situation [38].

An important insight provided by children from our needs assessment relates to norms that apply in the neighborhood, especially regarding unhealthy dietary behavior: drinking one can of Red Bull per day is considered fine and consuming healthy foods/drinks is considered as not ‘cool’. As this is the norm, and children and parents see it all around them, it is hard to change these behaviors. Even if children and parents know what is healthy and what is not, they do not have the skills to go against these norms. This finding indicates the importance of a more specific focus on intervention strategies targeting to change current norms. Previous general needs assessments also identified the consumption of sugared drinks as a determinant of childhood obesity but did not provide context-specific determinants [15]. However, knowledge of context-specific determinants can be essential for behavior change [39,40]. A systematic review on qualitative evidence for factors influencing unhealthy dietary behavior in young children supports our finding of the influence of social norms on dietary behavior [41]. For example, unhealthy modeling by parents but also by other family members and peers leads to unhealthy dietary choices. A big but crucial challenge is how to make a healthy diet cool or at least normal, so modeling of unhealthy dietary behavior diminishes.

Similarly, previous needs assessments identified a lack of physical activity as a potential determinant of childhood overweight/obesity [15,37]. Our needs assessment also identified this risk factor and our participatory approach additionally identified context-specific determinants of both the social and physical environment. In our study, and in previous research, the feeling of safety in the neighborhood has been found to have an effect on physical activity behaviors of youth [7]. In our needs assessment, we have further specified this, mostly acknowledging the soft drug shop and youth gang as influencing the feeling of unsafety. This feeling also relates to social cohesion in the neighborhood and looking out for each other. This has been found to influence physical activity of youth in previous research [42]. In our study, children also talked about this issue. They said for example that some children at their school did not like sports and that some children were too overweight and therefore did not participate in sports. To get these children to play outside or join sports, children mentioned that they should be invited by classmates, and activities should be supervised to prevent bullying. Additionally, parents also mentioned difficulties with changing their children’s behavior; children prefer videogames over playing outside, or it is not a habit of children—or their family—to be physically active. As with dietary behavior, modeling by parents has shown to be of influence on children’s physical activity behavior [43]. All of this information is essential when developing interventions aimed at promoting physical activity for this target group.

Involving a hard-to-reach group in the needs assessment may help to gain trust and to reach more community members. In the current project, children acted as co-researchers, and thereby gathered input from other children and family members who the researcher may not have been able to reach. Initially, no parents volunteered to participate in the planned interviews. As the researcher spent a lot of time in the community, the parents became more comfortable and shared their stories. Altogether, this resulted in a comprehensive, context-specific needs assessment. When children are also actively involved in the development and implementation of the interventions—i.e., participatory action research—and see their ideas becoming realized, this could result in reaching more children [22,26].

### Strengths and Limitations

The main strength of this participatory needs assessment is that we incorporated the voice of children and parents in research that applies to them, which is very valuable and helped this project towards a better understanding of the specific needs of the community. Children were not only involved in collecting data but also in its analysis, thereby increasing the validity of the results. Although we had no participatory meetings with parents, by participating in the neighborhood and talking to parents in their own environment we gained their trust and succeeded in involving them in the project. The collaboration with the municipality further strengthens our study, creating support in the community and getting in touch with schools. Having the municipality as a partner in this study at large may yield more sustainable interventions that are supported by relevant stakeholders. A limitation of participatory research is the generalizability of the results. Participatory research is, by definition, locally situated, meaning that the generated knowledge is context-specific and the findings should be interpreted accordingly. As this needs assessment is context-specific, it is not directly applicable to a different context. However, when comparing communities and finding similarities, findings could be transferable after cross-checking with the local community. Another limitation of this needs assessment is that we had limited time in the participatory group sessions with children, but by giving the children home assignments, the sessions were planned as effectively as possible. Because of the limited amount of participatory group sessions, it is hard to say if data saturation of the children’s perspective has been reached. However, the conclusions of all three participatory groups were comparable, suggesting data saturation. This is similar to the interviews with professionals. In total, 13 interviews were held and analyzed. As the interviews showed a lot of overlap, we assume data saturation. Meetings with parents were held till no new information was generated from the meetings. Another strength is that all data summaries were verified for accuracy by the respective stakeholder group.

## 5. Conclusions

Especially when focusing on a specific target group—i.e., a school, neighborhood—a participatory needs assessment can provide essential, in-depth context-specific insights. These insights may be essential in the development of interventions stimulating healthy behavior, as these interventions are more tailored to the specific health issues and needs of the target group. This study incorporated the perspectives of children, parents and professionals on the health issues and needs of children from one neighborhood. By involving the perspectives of these most relevant stakeholders, a comprehensive overview of the issues and possible effective solutions has been created.

## Figures and Tables

**Table 1:** Coding scheme.

Themes	Categories	Subcategories
Neighborhood characteristics	Safety	Youth gang Soft drug shop
	Overweight	Dietary behavior
		Lack of physical activity
	Language	
	Personality	Stubbornness Private
	Household characteristics	
	Finances	
	Culture	
Causes of overweight	Dietary behavior	Habit
		Knowledge/education
		Finances
		Marketing
		Culture
		Decisive power
	Lack of physical activity	Safety
		Habit
		Distance
		Finances
		Culture
		Health Knowledge
Ideas for solutions	Combine education with an activity	Cooking classes
	Approach	Participate with community Language
	Extra sports activities	Location
		Supervision
		Activities for girls
		Affordable
		Continuous program
